# Acute respiratory distress syndrome triggered by marked cytokine storm in a subject with diabetic ketoacidosis: A case report

**DOI:** 10.1097/MD.0000000000029119

**Published:** 2022-03-25

**Authors:** Megumi Horiya, Takatoshi Anno, Ryo Shigemoto, Katsumasa Koyama, Fumiko Kawasaki, Koichi Tomoda, Kohei Kaku, Hideaki Kaneto

**Affiliations:** a *Department of General Internal Medicine 1, Kawasaki Medical School, Okayama, Japan,*; b *Department of Diabetes, Endocrinology and Metabolism, Kawasaki Medical School, Kurashiki, Japan.*

**Keywords:** acute respiratory distress syndrome, cytokine storm, diabetic ketoacidosis, influenza infection, type 1 diabetes mellitus

## Abstract

**Rationale:**

Acute respiratory distress syndrome (ARDS) is an acute diffuse inflammatory lung injury. Many causes of acute direct and indirect lung injury have been described as possible initiators of ARDS. According to the literature data, ARDS could be a rare complication associated with the acute onset of diabetic ketoacidosis (DKA). Moreover, it has been suggested that cytokine release during DKA is involved in the above-mentioned acute clinical complications of DKA.

**Patientconcerns:**

A 48-year-old Japanese woman with a 4-year history of type 1 diabetes mellitus was brought to an emergency room with symptoms of deteriorated consciousness. Three days before, she was diagnosed with influenza A infection.

**Diagnosis:**

Inflammation markers were markedly elevated and she was under DKA condition. Since her respiratory conditions were suddenly and markedly aggravated 2 days later, we diagnosed her as ARDS and continued systemic management with the ventilator.

Interleukin-6 (IL-6) level was markedly elevated at the onset of ARDS, although IL-6 level was high at the onset of DKA. ARDS was suggested to be caused by marked cytokine storm and DKA.

**Interventions:**

We continued to treat her hyperglycemic crises. Moreover, we continued systemic management with the ventilator.

**Outcomes:**

Approximately three weeks later, her general conditions were stabilized and ventilator management was stopped. We successfully treated her ARDS and hyperglycemic crises.

**Lessons:**

This case is very important because it shows that DKA can induce cytokine storm, which leads to the onset of ARDS. Therefore, monitoring various cytokines such as IL-6, which are associated with ARDS during the period of treatment of DKA is beneficial.

## 1. Introduction

Acute respiratory distress syndrome (ARDS) is an acute diffuse inflammatory lung injury, clinically characterized by the sudden onset of dyspnea, progressive hypoxemia and decreased lung compliance accompanied by the development of diffuse infiltration on chest imaging.^[1,2]^ The pathogenesis of ARDS involves an inflammatory process at the alveolar-capillary interface, leading to the accumulation of protein-rich fluid in the alveolar space. Many causes of acute direct and indirect lung injury have been described as possible initiators of ARDS. According to the literature data, ARDS could be a rare complication associated with the acute onset of diabetic ketoacidosis (DKA), which usually occurs in adolescents and young adults.^[3-6]^

Diabetes mellitus (DM) is a chronic metabolic disorder caused by an absolute or relative deficiency of insulin. DKA is a serious acute metabolic complication of DM,^[7]^ and is often observed in subjects with type 1 DM (T1DM). Interstitial pulmonary edema has been reported to be present in children and adolescents with DKA before initiation of treatment.^[8]^ Moreover, it has been suggested that cytokine release during DKA is involved in the above-mentioned acute clinical complications of DKA in children with T1DM.^[9]^

## 2. Case presentation

**Figure 1.**
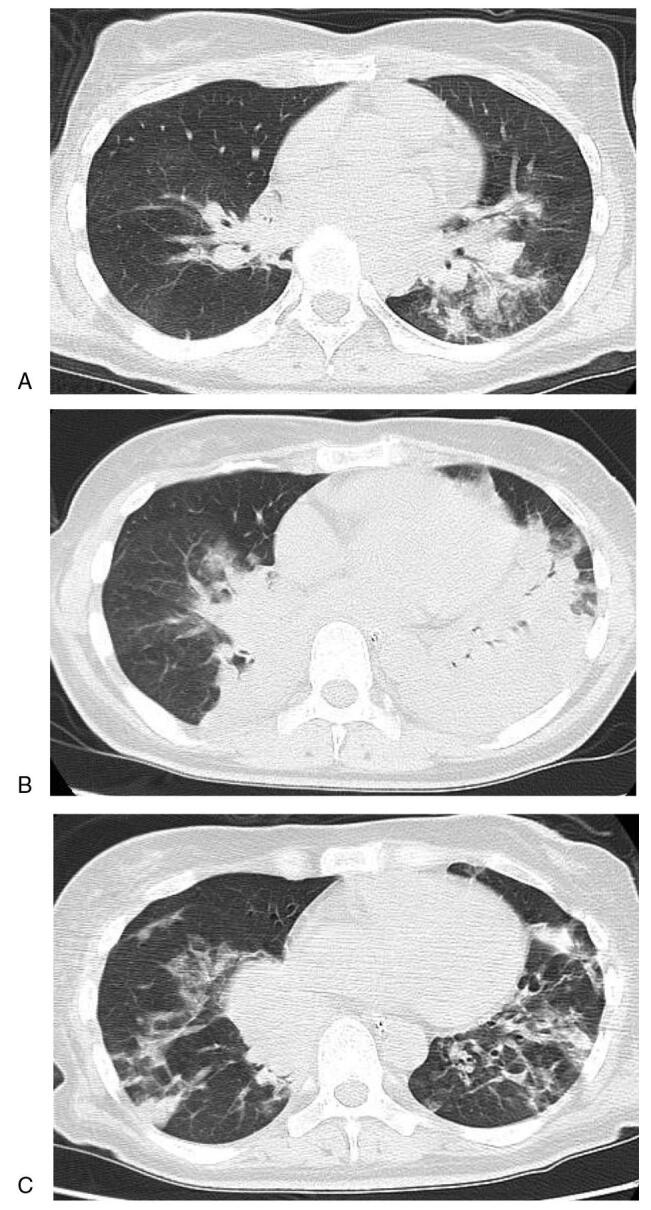
Chest computed tomography (CT) on admission (A), 2days after admission (B), 3weeks after admission (C). Chest CT on admission and at the onset of DKA showed pneumonia in the left lower lung. Chest CT taken at the onset of ARDS 2 days after admission showed bilateral dense-dependent consolidation mainly in the left lung. Chest CT taken 3weeks after admission showed consolidation shadow after pneumonia.

A 48-year-old Japanese woman with a 4-year history of T1DM was brought to an emergency room with symptoms of deteriorated consciousness. Three days before, she was diagnosed with influenza A infection, and treated with oseltamivir in the outpatient clinic. She was taking insulin therapy (18 unit/d of insulin aspart and 16unit/d of insulin glargine) in another hospital at that time. She had no remarkable past history and family history. Her height and body weight were 160.0cm and 64.5 kg, respectively. Her vital signs were as follows: temperature, not detected due to very low temperature; blood pressure, 96/42mm Hg; heart rate, 64beats/min; oxygen saturation, 98% under 10L of oxygen. Table 1 shows laboratory data in the emergency room. Inflammation markers were markedly elevated: white blood cell, 14,200/μL (neutrophil, 76.8%; lymphocyte, 18.7%); C-reactive protein, 4.16 mg/dL; procalcitonin, 1.64 ng/mL. Liver function was slightly elevated, and renal function was markedly elevated: aspartate aminotransferase, 69 U/L; alanine transaminase, 38 U/L; alkaline phosphatase, 263 U/L; γ-glutamyl transpeptidase, 7U/L; lactate dehydrogenase, 349U/L; creatinine, 2.34 mg/dL; blood urea nitrogen, 74mg/dL. Diabetesassociated data were as follows: plasma glucose, 801mg/dL; hemoglobin A1c, 13.3%; glycoalbumin 40.7%. Ketone body concentrations were markedly elevated: total ketone body, 15,980.0 μmol/L; acetoacetate, 1900.0 μmol/L; β-hydroxybuterate, 14,080.0 μmol/L. Blood gas analysis showed severe acidosis: pH 6.806. In addition, anti-glutamic acid decarboxylase antibody was markedly elevated up to 74,343.1 U/mL. As shown in Figure 1A, chest computed tomography on admission revealed pneumonia in the left lower lung. Based on such findings, we finally diagnosed her as pneumoniae after influenza A infection and DKA. Thus, we started the whole-body management against hypoxia and hyperglycemia.

**Table 1:** Laboratory data in an emergency room in this subject.

**Variable**	**Result**	**Reference range**
**Peripheral blood**		
White blood cells (/μL)	14280	3300-8600
Neutrophil (%)	76.8	52.0-80.0
Lymphocyte (%)	18.7	20.0-40.0
Red blood cells (×10^4^/μL)	578	386-492
Hemoglobin (g/dL)	13.6	11.6-14.8
Hematocrit (%)	46.7	35.1-44.4
Platelets (×10^4^/μL)	23.3	15.8-34.8
**Blood biochemistry**		
Total protein (g/dL)	7.0	6.6-8.1
Albumin (g/dL)	3.9	4.1-5.1
Globulin (g/dL)	3.1	2.2-3.4
Total bilirubin (mg/dL)	0.4	0.4-1.5
AST (U/L)	69	13-30
ALT (U/L)	38	7-23
LDH (U/L)	349	124-222
ALP (U/L)	263	106-322
γ-GTP (U/L)	7	9-32
BUN (mg/dL)	74	8-20
Creatinine (mg/dL)	2.34	0.46-0.79
Cholinesterase (U/L)	227	201-421
Uric acid (mg/dL)	15.8	2.6-5.5
Total cholesterol (mg/dL)	216	142-248
Sodium (mmol/L)	140	138-145
Potassium (mmol/L)	5.7	3.6-4.8
Chloride (mmol/L)	108	101-108
**Diabetes marker**		
Plasma glucose (mg/dL)	801	
Hemoglobin A1c (%)	13.3	4.9-6.0
Glycoalbumin (%)	40.7	12.4-16.3
Total ketone body (μmol/L)	15980.0	0.0-130.0
Acetoacetate (μmol/L)	1900.0	0.0-55.0
β-Hydroxybuterate (μmol/L)	14080.0	0.0-85.0
GAD Ab. (U/mL)	74343.1	< 5.0
**Infectious marker**		
CRP (mg/dL)	4.16	< 0.14
Procalcitonin (ng/mL)	1.64	0.00-0.05
**Blood Gas Analysis**		
pH	6.806	7.360-7.460
PCO_2_ (mm Hg)	19.2	34.0-46.0
PO_2_ (mm Hg)	199.0	80.0-90.0
HCO_3_^-^ (mEq/L)	2.9	24.0-32.0
BE (mEq/L)	-32.2	-2.5-2.5
SO_2_ (%)	99.1	95.0-98.0
Lactate (mEq/L)	1.40	0.63-2.44
**Urinary test**		
Urinary pH	5.0	5.0-7.5
Urinary protein	1+	-
Urinary sugar	3+	-
Urinary ketone body	3+	-
Urinary bilirubin	-	-
Urinary blood	3+	-

γ-GTP = γ-glutamyltranspeptidase, Ab. = antibody, ALP = alkaline phosphatase, ALT = alanine aminotransferase, AST = aspartate aminotransferase, BE = base excess, BUN = blood urea nitrogen, CRP = C-reactive protein, GAD = anti-glutamic acid decarboxylase, LDH = lactate dehydrogenase.

On admission in intensive care unit, we started to treat her hyperglycemic crises. We administered 0.9% NaCl and gradually tapered total volume. We also maintained serum potassium levels with drip infusion and continuous insulin infusion and gradually tapered continuous insulin. Her hyperglycemia and DKA conditions were improved. Moreover, we treated hypoxia, hypothermia and shock vital conditions. Two days later, her respiratory conditions were suddenly and markedly aggravated and she needed enough oxygenation with reservoir mask. Therefore, we intubated her and started the ventilator management. As shown Figure 1B, chest computed tomography taken 2 days after admission revealed bilateral dense-dependent consolidation mainly in the left lung, which was markedly aggravated compared to that on admission. We diagnosed her as ARDS, and we continued systemic management with the ventilator. Approximately three weeks later, her general conditions were stabilized and she was relieved from the ventilator management, although she suffered from continuous hypoxemia, bacterial and fungal pneumonia, rhabdomyolysis and electrolyte imbalance for 3 weeks. Finally, we successfully treated her ARDS and hyperglycemic crises (Fig. 1C). She was transferred from the intensive care unit to our general ward.

Since the conditions in this subject with ARDS were exacerbated very rapidly and just after the onset of DKA, we examined the cytokine levels during the disease course using residual serum to clarify the possible mechanism for DKA associated with ARDS. As shown in Table 2, interleukin-6 (IL-6) level was markedly elevated at the onset of ARDS, although IL-6 level was high at the onset of DKA. It clearly showed that ARDS was induced by cytokine storm after the onset of DKA. In addition, we believe elevated IL-6 level was, at least in part, induced by DKA, since IL-6 is an acute phase inflammatory marker that responds in a few hours.^[10]^

**Table 2:** Time course of various infection and diabetes markers just after the diagnosis of DKA and ARDS.

**Infection markers**		**Diagnosis of DKA in an emergency room**	**Diagnosis of ARDS 2 d after admission**	**Reference range**
White blood cell (/μL)		14,280	9920	3,500-8600
	Neutrophils (%)	76.8	88.0	52.0-80.0
	Lymphocytes (%)	18.7	10.0	20.0-40.0
C-reactive protein (mg/dL)		4.16	18.13	< 0.14
Procalcitonin (ng/mL)		1.64	5.29	0.00-0.05
**Diabetes markers**				**Reference range**
Plasma Glucose (mg/dL)		801	280
Total ketone body (μmol/L)		15,980.0	N/A	0.0-130.0
Acetoacetate (μmol/L)		1900.0	N/A	0.0-55.0
β-hydroxybuterate (μmol/L)		14,080.0	N/A	0.0-85.0
Urinary ketone body		3+	-	-
pH		6.806	7.360	7.360-7.460
Base excess (mEq/L)		-32.2	-5.3	-2.5-2.5
**Cytokines**				**Reference range**
IFN-γ (IU/mL)		N/A	< 0.1	< 0.1
TNF-a (pg/mL)		1.93	4.71	0.6-2.8
IL-1β (pg/mL)		< 10	< 10	< 10
IL-6 (pg/mL)		377	35,000	< 4.0
IL-8 (pg/mL)		2280	384	< 2.0

ARDS = acute respiratory distress syndrome, DKA = diabetic ketoacidosis, IFN-γ = interferon-γ, IL = interleukin, N/A = not applicable, TNF-α = tumor necrosis factor-α.

## 3. Discussion

Herein, we report a case of ARDS triggered by marked cytokine storm after DKA and influenza A infection in a subject with T1DM. The pathophysiology of association between ARDS and DKA remains uncertain, but there are few reports that ARDS triggered by marked cytokine storm under the treatment of DKA in adolescents.

Among the numerous physiologic responses requiring cytokine involvement, there are the development of cellular and humoral immune responses, the induction of the inflammatory response, regulation of hematopoiesis, and control of cellular proliferation and differentiation.^[11]^ Previously, it was reported that the serum concentration of IL-6 was elevated under hyperglycemic conditions.^[12,13]^ In addition, it was shown that levels of IL-6 together with tumor necrosis factor-a (TNF-α), were elevated in patients with DM and that inflammatory conditions led to the development of vascular disease and atherosclerosis probably by increasing oxidative stress.^[14-16]^ Moreover, it was reported that IL-6 level was significantly increased in T1DM subjects with long duration of diabetes as well as in subjects just after the onset of T1DM in children compared with healthy controls.^[9,17]^ On the other hand, IL-6 and other pro-inflammatory cytokines, such as TNF-α, IL-1β, and interferon-γ increase pulmonary permeability, resulting in pulmonary edema. In addition, previous studies support the idea that pro-inflammatory cytokines, especially IL-6, play a central role in the process of inflammation and endothelial damage.^[18,19]^ Thus, it is likely that ARDS is induced by cytokine storm and IL-6 plays a major role in the onset of ARDS.

Although there are some previous studies on the role of cytokines in the development of long-term diabetes complications, there is little information about the immunological response and fluctuation of cytokine levels during the metabolic crisis of DKA.^[20]^ Since this patient had T1DM, we think that her IL-6 and TNF-a levels were elevated at the onset of DKA. It is known that IL-6 is quickly released by inflammatory cells and Creactive protein production by hepatocytes requires a longer period.^[21]^ Therefore, we assume that her IL-6 level was markedly elevated mainly by inflammatory cells at the onset ARDS (377 to 35,000pg/mL) only 2 days after the onset of DKA. Therefore, we think that DKA was, at least in part, associated with the onset of ARDS triggered by inducing marked cytokine storm, although she suffered from influenza A infection as preceding infection.

Taken together, we should bear in mind the possibility that DKA after infectious disease causes cytokine storm with elevated IL-6 levels and that such cytokine storm leads to the onset of ARDS. We beleive this case is very important because it shows that DKA and/or viral infection can induce cytokine storm, which leads to the onset of ARDS. Therefore, we have to examine various cytokines such as IL-6, which are associated with ARDS during the period of treatment of DKA.

## Author contributions

T.A. is the guarantor of this work and, as such, had full access to all data in the study and takes responsibility for the integrity of the data and the accuracy. M.H., T.A., R.S., K.K. and F.K. researched the data and conducted inspections. M.H., T.A., R.S., K.K. and F.K. were in charge of treatment during hospitalization. K.T., K.K. and H.K. reviewed and edited the manuscript. K.T., K. K. and H.K. contributed to discussion.

**Conceptualization:** Takatoshi Anno.

**Data curation:** Megumi Horiya, Takatoshi Anno, Ryo Shigemoto, Katsumasa Koyama, Fumiko Kawasaki.

**Formal analysis:** Megumi Horiya, Takatoshi Anno, Ryo Shigemoto, Katsumasa Koyama, Fumiko Kawasaki.

**Investigation:** Megumi Horiya, Takatoshi Anno, Ryo Shigemoto, Katsumasa Koyama, Fumiko Kawasaki.

**Supervision:** Hideaki Kaneto.

**Visualization:** Takatoshi Anno.

**Writing** - **original draft:** Takatoshi Anno.

**Writing** - **review & editing:** Takatoshi Anno, Koichi Tomoda, Kohei Kaku, Hideaki Kaneto.
